# Truncated Power-Normal Distribution with Application to Non-Negative Measurements

**DOI:** 10.3390/e20060433

**Published:** 2018-06-05

**Authors:** Nabor O. Castillo, Diego I. Gallardo, Heleno Bolfarine, Héctor W. Gómez

**Affiliations:** 1 Departamento de Matemáticas, Facultad de Ciencias, Universidad de La Serena, La Serena 1700000, Chile; 2 Departamento de Matemática, Facultad de Ingeniería, Universidad de Atacama, Copiapó 1530000, Chile; 3 Departamento de Estatística, Instituto de Matemática e Estatística (IME), Universidade de São Paulo, São Paulo 01000-000, Brazil; 4 Departamento de Matemáticas, Facultad de Ciencias Básicas, Universidad de Antofagasta, Antofagasta 1240000, Chile

**Keywords:** maximum likelihood, power-normal distribution, truncation, Shannon entropy

## Abstract

This paper focuses on studying a truncated positive version of the power-normal (PN) model considered in Durrans (1992). The truncation point is considered to be zero so that the resulting model is an extension of the half normal distribution. Some probabilistic properties are studied for the proposed model along with maximum likelihood and moments estimation. The model is fitted to two real datasets and compared with alternative models for positive data. Results indicate good performance of the proposed model.

## 1. Introduction

Lehmann [1] proposed a class of asymmetric distributions. The cumulative distribution function (cdf) for such class is given by:

(1) $$F_{F}\left(z;\alpha \right)={\left\{F\left(z\right)\right\}}^{\alpha },\;\;\;\;z\in R,$$

where *F* is in itself a cumulative distribution function and α∈Q, with Q the set of rational numbers. In the special case where α is an integer number, the above cdf corresponds to the distribution of the maximum in a sample of size α.

Durrans [2] gives an interpretation for (1) in the more general case $\alpha \in R^{+}$ based on *fractional order statistics*. Assume *F* is an absolutely continuous function and *f* denotes its respective probability density function (pdf), i.e., f=dF. The pdf related to (1) is:

(2) $$f_{F}\left(z;\alpha \right)=\alpha f\left(z\right){\left\{F\left(z\right)\right\}}^{\alpha -1},\;\;\;\;\;\;z\in R,\;\;\alpha \in R^{+}.$$

Henceforth, we refer to a random variable with pdf as in (2) as the *power distribution ($P_{F}$)*, and we use the notation $Z∼P_{F}\left(\alpha \right)$. The particular case where F=Φ(·), the cdf of the standard normal model, was approached in [2]. In such a case, the respective pdf of the model is reduced to:

(3) $$f_{\Phi }\left(z;\alpha \right)=\alpha \phi \left(z\right){\left\{\Phi \left(z\right)\right\}}^{\alpha -1},\;\;\;\;z\in R,\;\;\alpha \in R^{+},$$

where ϕ(·) is the standard normal pdf. The authors used the term *generalized Gaussian* distribution to refer the model in Equation (3). This model also was studied with more detail by [3]. Pewsey et al. [4] call Model (3) the *power-normal (PN) model*, denoting Z∼PN(α), and show that its Fisher information matrix (FIM) for the location-scale extension is nonsingular for α=1 (i.e., the symmetric case).

The generalization of the normal distribution in (3) also is a particular case of the Beta-normal model discussed in [5].

On the other hand, the random variable *X* follows a half-normal distribution with scale parameter σ if its pdf is given by:

$$f_{HN}\left(x;\sigma \right)=\frac{2}{\sigma }\phi \left(\frac{x}{\sigma }\right)I\left\{x>0\right\},$$

for σ>0. We denote X∼HN(σ). Cooray and Ananda [6] extended the half-normal (HN) model by introducing the generalized half-normal (GHN) model, that is *X* is a random variable with the GHN distribution with scale parameter σ and shape parameter α, if its pdf is given by:

$$f_{GHN}\left(x;\sigma ,\alpha \right)=\sqrt{\frac{2}{\pi }}\left(\frac{\alpha }{x}\right){\left(\frac{x}{\sigma }\right)}^{\alpha }\exp\left[-\frac{1}{2}{\left(\frac{x}{\sigma }\right)}^{2\alpha }\right]I\left\{x>0\right\}\;,\;\;\sigma >0,\alpha >0.$$

We use the notation X∼GHN(σ,α). Observe that GHN(σ,α=1)≡HN(σ), that is one obtains the half-normal model with scale parameter σ>0.

Some properties of the GHN distribution are:

1. $H\left(x;\sigma ,\alpha \right)=2\Phi \left({\left(\frac{x}{\sigma }\right)}^{\alpha }\right)-1$
2. $E\left(X\right)=\sqrt{\frac{2^{1/\alpha }}{\pi }}\Gamma \left(\frac{1+\alpha }{2\alpha }\right)\sigma$
3. $Var\left(X\right)=\frac{2^{1/\alpha }}{\pi }\left(\sqrt{\pi }\Gamma (\frac{2+\alpha }{2\alpha })-\Gamma^{2}\left(\frac{1+\alpha }{2\alpha }\right)\right)\sigma^{2}$
4. $E\left(X^{r}\right)=\sqrt{\frac{2^{r/\alpha }}{\pi }}\Gamma \left(\frac{r+\alpha }{2\alpha }\right)\sigma^{r}$, for r=1,2,…,

where H(·) is the cdf of *X* and Γ(·) is the gamma function. The proofs of those properties are presented in [6]. Recent extensions of the HN model are considered in [7,8], among others.

The recent literature has experienced a growth in the theory and applications of the continuous truncated models. Among others, we refer the reader to [9,10,11,12,13,14,15,16].

The main focus of this paper is to study the positive truncation for the model considered in (3), where the normalizing constant for the pdf (3) is to be determined, and the resulting model is an extension of the half-normal distribution. That is, we generate a more flexible extension of the half-normal distribution that we call the truncated positive power-normal (TPN) distribution, where the asymmetry parameter α is a shape parameter. Given its flexibility, the model is quite useful for fitting positive data related to survival analysis and reliability.

The paper is organized as follows. In Section 2, we present the TPN distribution. Some basic properties such as the quantile function, the risk function and some moments are considered, and Shannon entropy is studied. In Section 3, we discuss some inferential aspects such as the log-likelihood function and its maximization, the corresponding Fisher information matrix (FIM) and the method of moments estimation. Section 4 deals with an extension of the TPN model and presents results for a small-scale simulation study, indicating good parameter recovery. Results of using the proposed model in two real applications are reported in Section 5. The main conclusion is that the TPN model can be a viable alternative for adjusting positive data.

## 2. The Truncated Positive PN Distribution

In this section, we present the pdf of the TPN model, some of its basic properties, moments and asymmetry and kurtosis coefficients.

### 2.1. The Probability Density Function

**Proposition** **1.** *A random variable Z has a TPN distribution and is denoted as Z∼TPN(σ;α) with parameters σ and α, if its pdf is given by:*

(4) $$f_{Z}\left(z;\sigma ,\alpha \right)=\frac{2^{\alpha }\alpha }{(2^{\alpha }-1)\sigma }\phi \left(\frac{z}{\sigma }\right){\left\{\Phi \left(\frac{z}{\sigma }\right)\right\}}^{\alpha -1}I\left\{z>0\right\},\;\;\;\;\;\;\sigma ,\alpha \in R^{+}.$$

**Proof.**  Under the assumption that X∼PN(σ,α), the pdf for the model TPN follows after computing the conditional distribution of Z=X|X>0, concluding the proof. ☐

**Remark** **1.** *For α∈N={1,2,…}, the TPN model admits the following stochastic representation. If $W_{1},\ldots ,$ are independent and identically distributed (iid) random variables with common distribution $N(0,\sigma^{2})$, then:*

$$Z=\max\left(0,X_{1},\ldots ,X_{\alpha }\right)∼TPN\left(\sigma ,\alpha \right).$$

Its distribution function is given by:

(5) $$F_{Z}\left(z;\sigma ,\alpha \right)=\left(\frac{2^{\alpha }}{2^{\alpha }-1}\right)\left(\Phi^{\alpha }\left(\frac{z}{\sigma }\right)-\frac{1}{2^{\alpha }}\right),$$

**Figure 1:**
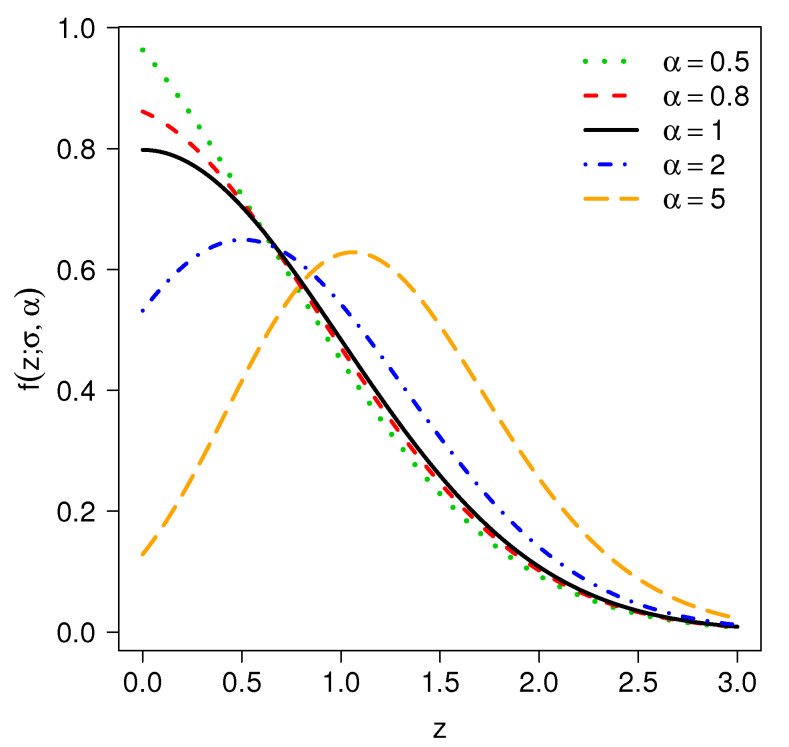
Probability density function of TPN(α,σ=1) for different values of α.

For σ=1 and varying α, Figure 1 depicts examples of the pdf for model TPN.

### 2.2. Properties

#### 2.2.1. Quantile Function

Simple algebraic manipulations yield:

$$Q\left(p\right)=\sigma \Phi^{-1}\left({\left(\frac{p(2^{\alpha }-1)+1}{2^{\alpha }}\right)}^{1/\alpha }\right),$$

for a probability 0<p<1. The quartiles are, consequently:

1. First quartile = $\sigma \Phi^{-1}\left({\left(\frac{2^{\alpha }+3}{2^{\alpha +2}}\right)}^{1/\alpha }\right)$
2. Median(Z) = $\sigma \Phi^{-1}\left({\left(\frac{2^{\alpha }+1}{2^{\alpha +1}}\right)}^{1/\alpha }\right)$
3. Third quartile = $\sigma \Phi^{-1}\left({\left(\frac{3(2^{\alpha }-1)+4}{2^{\alpha +2}}\right)}^{1/\alpha }\right)$

#### 2.2.2. Hazard Rate Function

The hazard rate function for the random variable Z∼TPN(σ,α) is given by:

$$h\left(z\right)=\frac{f_{Z}\left(z\right)}{1-F_{Z}\left(z\right)}=\frac{\alpha \phi \left(\frac{z}{\sigma }\right)\Phi^{\alpha -1}\left(\frac{z}{\sigma }\right)}{\sigma \left(1-\Phi^{\alpha }\left(\frac{z}{\sigma }\right)\right)},$$

**Remark** **2.**

*(i)*  *If α=1, then h(z) is the hazard function for the half-normal model $\forall z\in R^{+}$.*
*(ii)*  *$\forall \sigma ,\alpha ,z\in R^{+}$, h(z) is monotonically increasing with $h\left(0\right)=\sqrt{\frac{2}{\pi }}\frac{\alpha }{\sigma (2^{\alpha }-1)}$.*
*(iii)*  *∀σ,α, h(z)→∞, as z→∞.*

**Figure 2:**
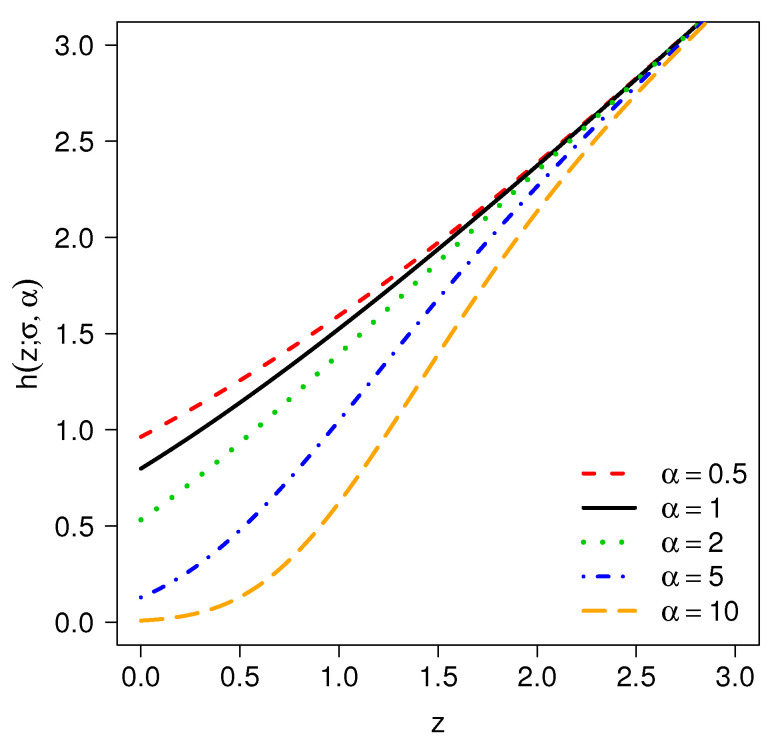
Hazard rate function of the TPN(α,σ=1) model and different values for α.

For σ=1 and varying α, Figure 2 depicts examples of the hazard rate function for model TPN.

### 2.3. Moments

**Proposition** **2.** *If Z∼TPN(σ,α), then the r-th moment of Z is:*

$$\mu_{r}=E\left(Z^{r}\right)=\frac{\alpha 2^{\alpha }\sigma^{r}}{2^{\alpha }-1}d_{r}\left(\alpha \right),\;\;\;\;\;\;\;\;r=1,2,\ldots$$

*where $d_{r}\left(\alpha \right)=\int_{1/2}^{1}{\left(\Phi^{-1}\left(u\right)\right)}^{r}u^{\alpha -1}du$ has to be computed numerically.*

**Proof.**  Making the variable change, $u=\Phi \left(\frac{z}{\sigma }\right)$, we obtain:

$$E\left(Z^{r}\right)=\int_{0}^{\infty }\frac{2^{\alpha }\alpha z^{r}}{(2^{\alpha }-1)\sigma }\phi \left(\frac{z}{\sigma }\right){\left\{\Phi \left(\frac{z}{\sigma }\right)\right\}}^{\alpha -1}dz=\int_{1/2}^{1}\frac{2^{\alpha }\alpha \sigma^{r}}{\left(2^{\alpha }-1\right)}{\left(\Phi^{-1}\left(u\right)\right)}^{r}u^{\alpha -1}du.$$

 ☐

**Corollary** **1.** *Therefore, the first four moments are given by:*

*(a)*  $\mu_{1}=E\left(Z\right)=\frac{\alpha 2^{\alpha }\sigma }{2^{\alpha }-1}d_{1}\left(\alpha \right).$
*(b)*  $\mu_{2}=E\left(Z^{2}\right)=\frac{\alpha 2^{\alpha }\sigma^{2}}{2^{\alpha }-1}d_{2}\left(\alpha \right).$
*(c)*  $\mu_{3}=E\left(Z^{3}\right)=\frac{\alpha 2^{\alpha }\sigma^{3}}{2^{\alpha }-1}d_{3}\left(\alpha \right).$
*(d)*  $\mu_{4}=E\left(Z^{4}\right)=\frac{\alpha 2^{\alpha }\sigma^{4}}{2^{\alpha }-1}d_{4}\left(\alpha \right).$

**Corollary** **2.** *Asymmetry and kurtosis coefficients are given, respectively, by:*

$$\sqrt{\beta_{1}}=\frac{{\left(2^{\alpha }-1\right)}^{2}d_{3}\left(\alpha \right)-3\alpha \left(2^{\alpha }-1\right)2^{\alpha }d_{1}\left(\alpha \right)d_{2}\left(\alpha \right)+\alpha^{2}2^{2\alpha +1}{\left[d_{1}\left(\alpha \right)\right]}^{3}}{\sqrt{\alpha 2^{\alpha }}{\left[\left(2^{\alpha }-1\right)d_{2}\left(\alpha \right)-\alpha 2^{\alpha }{\left[d_{1}\left(\alpha \right)\right]}^{2}\right]}^{3/2}}$$

*and:*

$$\beta_{2}=\frac{{\left(2^{\alpha }-1\right)}^{3}d_{4}\left(\alpha \right)-\alpha {\left(2^{\alpha }-1\right)}^{2}2^{\alpha +2}d_{1}\left(\alpha \right)d_{3}\left(\alpha \right)+3\alpha^{2}2^{2\alpha +1}\left(2^{\alpha }-1\right){\left[d_{1}\left(\alpha \right)\right]}^{2}d_{2}\left(\alpha \right)-3\alpha^{3}2^{3\alpha }{\left[d_{1}\left(\alpha \right)\right]}^{4}}{\alpha 2^{\alpha }{\left[\left(2^{\alpha }-1\right)d_{2}\left(\alpha \right)-\alpha 2^{\alpha }{\left[d_{1}\left(\alpha \right)\right]}^{2}\right]}^{2}}.$$

**Figure 3:**
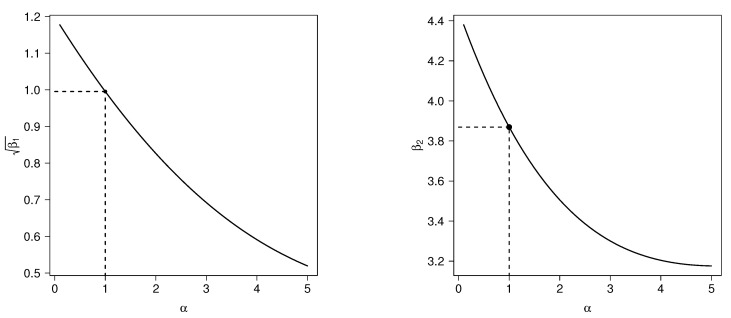
Asymmetry (**left**) and kurtosis (**right**) coefficients for TPN(σ,α) (solid line) and half normal (HN) (α=1, dotted line).

**Remark** **3.** *If α=1, the asymmetry and kurtosis coefficients take the values 0.99527 and 3.86918, respectively, which correspond to those for the classical HN distribution. Figure 3 depict plots for the asymmetry and kurtosis coefficients, respectively, of the HN and TPN distribution.*

### 2.4. Shannon Entropy

Shannon entropy (see [17]) measures the amount of uncertainty for a random variable *Z*. It is defined as:

$$S\left(Z\right)=-E(\log f_{Z}\left(z\right)).$$

Therefore, it can be verified that the Shannon entropy for the TPN model is:

(6) $$S\left(Z\right)=1-\frac{1}{\alpha }+\log\left(\frac{\sigma }{\alpha }\right)+\log\left(2^{\alpha }-1\right)+\frac{1}{2}\log\left(2\pi \right)+\frac{\alpha 2^{\alpha -1}d_{2}\left(\alpha \right)}{2^{\alpha }-1}-\left[\alpha +\frac{\alpha -1}{2^{\alpha }-1}\right]\log\left(2\right),$$

**Figure 4:**
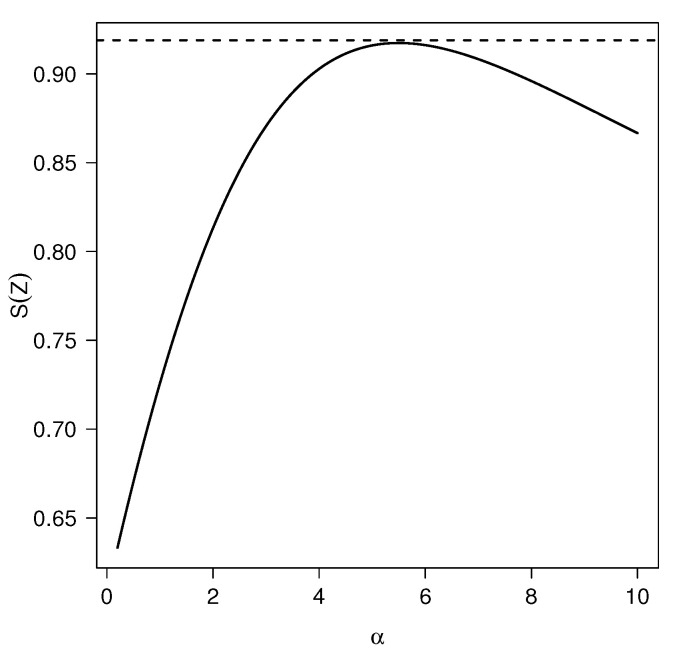
Shannon entropy of TPN(α,σ=1) for different values of α. The dashed line corresponds to the Shannon entropy for the standard normal model.

Figure 4 shows the Shannon entropy for the TPN model fixing σ=1. Note that for a fixed σ, S(Z) is maximized at α≈5.4962.

**Remark** **4.**

*(i)*  *From Figure 4 and for a fixed σ, we conclude that $S\left(Z\right)\leq S^{N}\left(Z\right)$, ∀α>0, where $S^{N}\left(Z\right)$ denotes the Shannon entropy for the $N(0,\sigma^{2})$ distribution.*
*(ii)*  *For α=1, it follows that $d_{2}\left(1\right)=1/2$, and S(Z) agrees with the entropy for the half-normal distribution (see [18]), which is given by:*
  $$S^{HN}\left(Z\right)=\frac{1}{2}+\frac{1}{2}\log\left(\frac{\pi \sigma^{2}}{2}\right).$$

### 2.5. Rényi Entropy

A generalization of the Shannon entropy is the Rényi entropy, which is defined as:

$$R_{p}\left(Z\right)=\frac{1}{1-p}\log\left(\int_{0}^{\infty }{\left[f\left(z\right)\right]}^{p}dz\right).$$

Routine calculations show that for the TPN model:

$$\begin{array}{cc} R_{p}\left(Z\right) & =\log\left(\sqrt{2\pi }\sigma \right)+\frac{p}{1-p}\left(\alpha \log\left(2\right)+\log\alpha -\log\left(2^{\alpha }-1\right)\right)-\frac{1}{2(1-p)}\log\left(p\right)\\ & \;\;\;\;+\frac{1}{\left(1-p\right)}\int_{0}^{\infty }\phi \left(w\right){\left\{\Phi \left(\sqrt{p}w\right)\right\}}^{p(\alpha -1)}dw. \end{array}$$

**Remark** **5.** *For m=p(α−1)∈N={1,2,…,}, the $R_{p}\left(Z\right)$ is reduced to:*

$$R_{p}\left(Z\right)=\log\left(\sqrt{2\pi }\sigma \right)+\frac{p}{1-p}\left(\alpha \log\left(2\right)+\log\alpha -\log\left(2^{\alpha }-1\right)\right)-\frac{1}{2(1-p)}\log\left(p\right)+\frac{1}{\left(1-p\right)c_{m}\left(\sqrt{p}\right)},$$

*where $c_{m}\left(\sqrt{p}\right)$ is the normalization constant in the Balakrishnan skew-normal distribution ([19,20]). In the last two references, the following facts are shown:*

*(a)*  *${\left[c_{1}\left(\sqrt{p}\right)\right]}^{-1}=\frac{1}{2}$.*
*(b)*  *${\left[c_{2}\left(\sqrt{p}\right)\right]}^{-1}=\frac{1}{4}+\frac{1}{2\pi }\sin^{-1}\left(\frac{p}{1+p}\right)$.*
*(c)*  *${\left[c_{3}\left(\sqrt{p}\right)\right]}^{-1}=\frac{1}{8}+\frac{3}{4\pi }\sin^{-1}\left(\frac{p}{1+p}\right)$.*
*(d)*  *∀m∈N, ${\left[c_{m}\left(\sqrt{p}\right)\right]}^{-1}\to \frac{1}{2}$, for $\sqrt{p}\to \infty$.*
*(e)*  *For m≥4, there is no closed form expression for ${\left[c_{m}\left(\sqrt{p}\right)\right]}^{-1}$. However, approximated values are provided in Table 1 from [21].*

### 2.6. Kullback–Leibler Divergence for HN and TPN Models

The Kullback–Leibler divergence ($D_{KL}\left(f_{1},f_{2}\right)$) is a measure of how one pdf (say $f_{1}$) diverges from a second (say $f_{2}$) pdf. For this reason, it can be used as a measure to decide between two alternative models for a particular dataset. As the HN model is a particular case of the TPN model (for α=1), we compute the Kullback–Leibler from the $HN(\sigma_{1})$ and $TPN(\sigma_{2},\alpha )$ models, which can be shown to be given by:

$$\begin{array}{cc} D_{KL}\left(TPN,HN\right) & =-\int_{0}^{\infty }\log\left(\frac{f_{HN}\left(z;\sigma_{1}\right)}{f_{TPN(z;\sigma_{2},\alpha )}}\right)f_{TPN}\left(z;\sigma_{2},\alpha \right)dz\\ & =-\frac{1}{2}\log\left(\frac{2}{\pi }\right)+\log\sigma_{1}+\frac{1}{2\sigma_{1}^{2}}E_{\sigma_{2},\alpha }\left(Z^{2}\right)-S\left(Z\right)\\ & =\frac{1}{\alpha }-1+\log\left(\frac{\sigma_{1}\alpha }{\sigma_{2}}\right)+\frac{\alpha 2^{\alpha -1}d_{2}\left(\alpha \right)}{2^{\alpha }-1}\left(\frac{\sigma_{2}^{2}}{\sigma_{1}^{2}}-1\right)-\log\left(2^{\alpha }-1\right)+\left(\alpha -1\right)\left[1+\frac{1}{2^{\alpha }-1}\right]\log\left(2\right). \end{array}$$

**Remark** **6.** *As expected, $D_{KL}\left(TPN,HN\right)=0$, if $\sigma_{1}=\sigma_{2}$ and α=1.*

## 3. Inference

In this section, we discuss moments and maximum likelihood estimation (MLE) and FIM and present a simulation study to investigate parameter recovery.

### 3.1. Moments Estimation

Solving for σ in Equation (a) from Corollary 1 and replacing $\bar{Z}$ for E(Z), it follows that:

(7) $$\begin{array}{ccc} \sigma & = & \frac{\left(2^{\alpha }-1\right)\bar{Z}}{\alpha 2^{\alpha }d_{1}\left(\alpha \right)}, \end{array}$$

Thus, replacing σ, given in Equation (8), and the second sample moment in Equation (b) from Corollary 1, it follows that:

(8) $$\begin{array}{c} \bar{Z^{2}}\alpha 2^{\alpha }{\left[d_{1}\left(\alpha \right)\right]}^{2}-\left(2^{\alpha }-1\right){\bar{Z}}^{2}d_{2}\left(\alpha \right)=0 \end{array}$$

Solving the equation given in (9) for α, we obtain ${\hat{\alpha }}_{M}$, and hence, replacing α by ${\hat{\alpha }}_{M}$ in Equation (8), one obtains ${\hat{\sigma }}_{M}$. This leads to the moments’ estimators $\left({\hat{\sigma }}_{M},{\hat{\alpha }}_{M}\right)$ for $\left(\sigma ,\alpha \right)$. The equation given in (9) is solved numerically using the function solve available in the software MAPLE.

### 3.2. The Log-Likelihood Function

For a random sample $Z_{1},\ldots ,Z_{n}$ from the distribution TPN(σ,α), the log likelihood function can be written as:

(9) $$l\left(\sigma ,\alpha \right)=n\left(\log\left(\frac{\alpha }{\sqrt{2\pi }\sigma }\right)-\log\left(1-2^{-\alpha }\right)\right)-\frac{1}{2\sigma^{2}}\sum_{i=1}^{n}z_{i}^{2}+\left(\alpha -1\right)\sum_{i=1}^{n}\log\left[\Phi \left(\frac{z_{i}}{\sigma }\right)\right],$$

so that the likelihood equations are given by:

(10) $$\begin{array}{ccc} \frac{1}{\sigma^{3}}\sum_{i=1}^{n}z_{i}^{2}-\frac{\left(\alpha -1\right)}{\sigma^{2}}\sum_{i=1}^{n}\frac{z_{i}\phi \left(\frac{z_{i}}{\sigma }\right)}{\Phi \left(\frac{z_{i}}{\sigma }\right)} & = & \frac{n}{\sigma } \end{array}$$

(11) $$\begin{array}{ccc} \frac{\log(2)}{2^{\alpha }-1}+\sum_{i=1}^{n}\log\left[\Phi \left(\frac{z_{i}}{\sigma }\right)\right] & = & \frac{n}{\alpha } \end{array}$$

The solution for Equations (10)–(11) can be obtained by using the function optim available in [22], and the specific method is the L-BFGS-B developed by [23], which allows constrained optimization, which uses a limited-memory modification of the quasi-Newton method.

#### Fisher Information Matrix

Let random variable Z∼TPN(σ,α). For a single observation *z*, the log-likelihood function for θ=(σ,α) is:

$$\log f_{Z}\left(\theta ;z\right)=\log\left(\alpha \right)-\log\left(\sigma \right)+\alpha \log\left(2\right)-\log\left(2^{\alpha }-1\right)-\log\left(\sqrt{2\pi }\right)-\frac{z^{2}}{2\sigma^{2}}+\left(\alpha -1\right)\log\left[\Phi \left(z/\sigma \right)\right]$$

The first derivatives of $\log f_{Z}\left(\theta ,z\right)$ are:

$$\begin{array}{ccc} \frac{\partial \log f_{Z}\left(\theta ;z\right)}{\partial \sigma } & = & -\frac{1}{\sigma }+\frac{z^{2}}{\sigma^{3}}-\left(\alpha -1\right)\frac{z}{\sigma^{2}}\frac{\phi (z/\sigma )}{\Phi (z/\sigma )}\\ \frac{\partial \log f_{Z}\left(\theta ;z\right)}{\partial \alpha } & = & \frac{1}{\alpha }+\log\left(2\right)-\frac{2^{\alpha }\log\left(2\right)}{2^{\alpha }-1}+\log\left[\Phi \left(z/\sigma \right)\right] \end{array}$$

The second derivatives of $\log f_{Z}\left(\theta ,z\right)$ are:

$$\begin{array}{ccc} \frac{\partial^{2}\log f_{Z}\left(\theta ;z\right)}{\partial \sigma^{2}} & = & \frac{1}{\sigma^{2}}-\frac{3z^{2}}{\sigma^{4}}-\frac{z(\alpha -1)}{\sigma^{3}}\left(\frac{z^{2}}{\sigma^{2}}\frac{\phi (z/\sigma )}{\Phi (z/\sigma )}+\frac{z}{\sigma }{\left(\frac{\phi (z/\sigma )}{\Phi (z/\sigma )}\right)}^{2}-2\frac{\phi (z/\sigma )}{\Phi (z/\sigma )}\right)\\ \frac{\partial^{2}\log f_{Z}\left(\theta ;z\right)}{\partial \alpha^{2}} & = & -\frac{1}{\alpha^{2}}+\frac{2^{\alpha }{\left(\log\left(2\right)\right)}^{2}}{{\left(2^{\alpha }-1\right)}^{2}}\\ \frac{\partial^{2}\log f_{Z}\left(\theta ;z\right)}{\partial \sigma \partial \alpha } & = & -\frac{z}{\sigma^{2}}\frac{\phi (z/\sigma )}{\Phi (z/\sigma )} \end{array}$$

It can be shown that the FIM for the TPN distribution is given by:

$$I_{F}\left(\sigma ,\alpha \right)=\left(\begin{array}{cc} I_{\sigma \sigma } & I_{\sigma \alpha }\\ I_{\sigma \alpha } & I_{\alpha \alpha } \end{array}\right)$$

with the following elements:

$$\begin{array}{ccc} I_{\sigma \sigma } & = & -\frac{1}{\sigma^{2}}+\frac{3}{\sigma^{4}}a_{20}+\frac{\left(\alpha -1\right)}{\sigma^{3}}\left(\frac{1}{\sigma^{2}}a_{31}+\frac{1}{\sigma }a_{22}-2a_{11}\right)\\ I_{\sigma \alpha } & = & \frac{1}{\sigma^{2}}a_{11}\\ I_{\alpha \alpha } & = & \frac{1}{\alpha^{2}}-\frac{2^{\alpha }{\left(\log\left(2\right)\right)}^{2}}{{\left(2^{\alpha }-1\right)}^{2}}, \end{array}$$

where $a_{ij}=E\left[Z^{i}{\left(\frac{\phi (z/\sigma )}{\Phi (z/\sigma )}\right)}^{j}\right]$, for i,j=1,2,3, must be computed numerically.

### 3.3. Truncation at c

As the following result indicates, the truncation point for the distribution TPN can be located at any c≥0. We denote this extension by $Z∼TPN_{c}$.

**Proposition** **3.** *A random variable $Z∼TPN_{c}$, if its pdf is given by:*

(12) $$f_{Z}\left(z;\sigma ,\alpha ,c\right)=\frac{\alpha }{(1-\Phi^{\alpha }\left(c/\sigma \right))\sigma }\phi \left(\frac{z}{\sigma }\right)\Phi^{\alpha -1}\left(\frac{z}{\sigma }\right)I\left\{z>c\right\},\;\;\;\;\;\;\sigma ,\alpha \in R^{+}$$

*where ϕ(·) and Φ(·) denote the pdf and cdf of the standard normal distribution, respectively. We use the notation $Z∼TPN_{c}\left(\sigma ,\alpha \right)$.*

**Proof.**  Under the assumption that X∼PN(σ,α), the pdf for the model TPN arises after computing the conditional distribution of Z=X|X>c, concluding the proof. ☐

## 4. Simulation Study

In this section, we present a brief simulation study in order to assess the performance of the MLEs of the TPN model in finite samples. To simulate from the $TPN_{c}$ distribution, it is sufficient to simulate from the PN distribution, accepting only those values greater than *c*. The simulation algorithm is then:

1. Simulate U∼U(0,1), and compute $Y=\sigma \Phi^{-1}\left(U^{1/\alpha }\right)$.
2. If Y≥c, make Z=Y. Otherwise, go to the previous step.

The acceptance ratio is then $1-\Phi^{\alpha }\left(c/\sigma \right)$. Hereafter, *c* is considered known and taking values of 0, 0.5 and 1.0. Likewise, for α and σ were chosen three values, and the generated samples were of sizes n=30, n=50, n=100 and n=200. For each combination of sample size and parameter values, 1000 samples were generated and MLEs were computed. Table 1 and Table 2 summarize the mean of the estimated parameters (mean), the mean of the estimated standard deviations (s.d.) and the root of the mean squared error ($\sqrt{MSE}$). Note that a small sample size (say n=30 and n=50) presents a moderate bias for both parameters, which are decreasing for *n* increasing. Additionally, the s.d.’s are closer to $\sqrt{MSE}$, especially when *n* is increased, suggesting that the s.d. are well estimated even in small sample sizes.

### Examples

**Figure 5:**
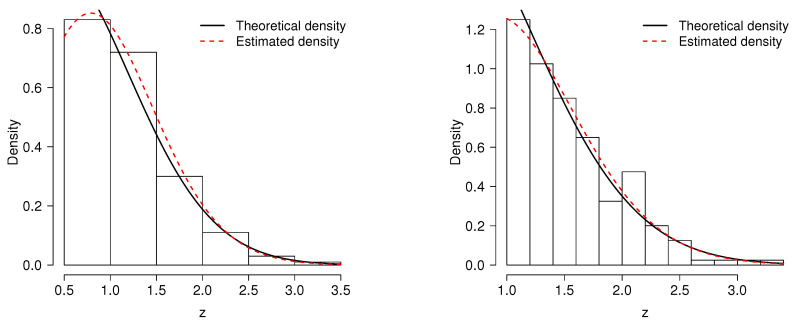
Examples of the estimation of the ${\mathrm{TPN}}_{c}\left(\sigma =1.0,\alpha =1.5\right)$ with their corresponding estimates. **Left panel**: c=0.5, $\hat{\alpha }=1.695$ and $\hat{\sigma }=1.032$. **Right panel**: c=1.0, $\hat{\alpha }=1.578$ and $\hat{\sigma }=0.959$.

Figure 5 depicts the model fitting for some simulated samples of size n=200 and truncations at c=0.5 and at c=1.

## 5. Real Data Illustration

In this section, we present two applications to illustrate the performance of the TPN model compared with other usual distributions in the literature, such as the Weibull, gamma, GHN, Birnbaum–Saunders (BS, [24,25]), β-Birnbaum–Saunders (β-BS, [26]), epsilon half-normal (EHN, [7]), power half-normal (PHN, [27]) and truncated positive normal (TN, [28]) models. Model comparison is implemented by using the AIC ([29]).

### 5.1. Australian Athletes

This dataset consists of several variables recorded on 202 Australian athletes and reported in [30]. Concretely, we analyze here measurements of the body mass index (BMI). Table 3 presents basic descriptive statistics for the dataset. We use the notation $\sqrt{b_{1}}$ and $b_{2}$ to represent sample asymmetry and kurtosis coefficients, respectively.

Using results from Section 3.1, moment estimators were computed leading to the following values: ${\hat{\sigma }}_{M}=7.644$ and ${\hat{\alpha }}_{M}=447.867$, which were used as initial estimates for the maximum likelihood (ML) approach. In this case, we fixed two values for the TPN model, namely c=0 and c=16 (a value close to the sample minimum).

**Figure 6:**
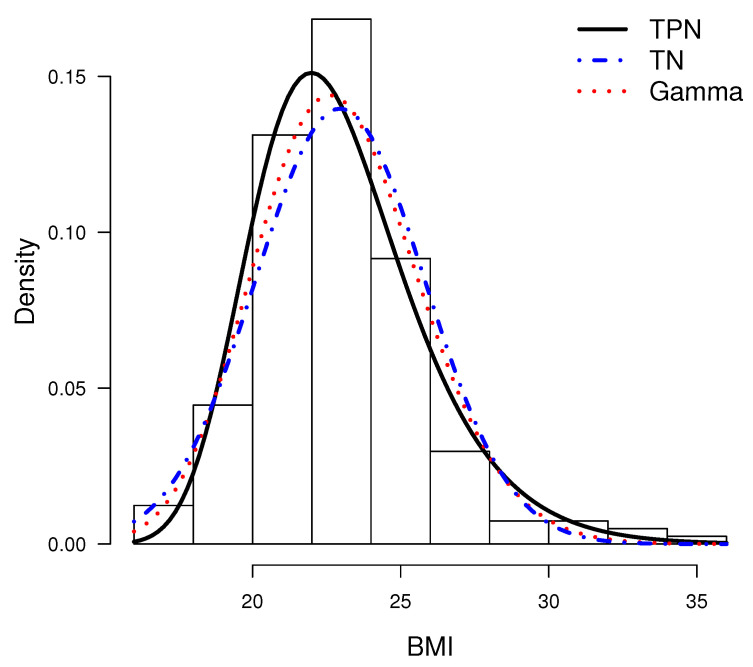
Histogram for the BMI dataset, with lines representing adjusted distributions using MLE for different models.

**Figure 7:**
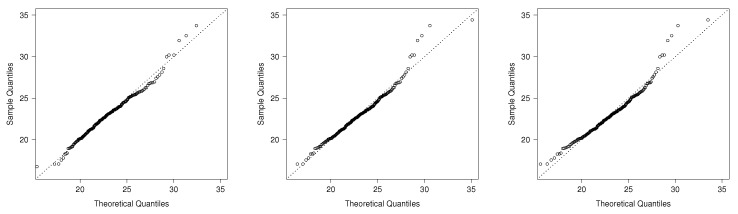
q-q plots: TPN model (**left**), gamma model (**center**) and TN model (**right**).

Table 4 depicts parameters’ estimates by maximum likelihood using the **bbmle** function in [22]. The standard errors of the MLE are calculated using the information matrix of each model. For each, we report the estimated log-likelihood function and the corresponding AIC. It can be noted that the AIC scores indicate better fit of the TPN model. On the other hand, results for c=0 and c=16 are similar. Therefore, we chose the standard model with c=0. In Figure 6, the estimated densities of the models using the ML estimates are shown with the data histogram. This also indicates good fit for the TPM model. Finally, Figure 7 shows the q-q plots for the TPN model and the other considered models. Note that TPN is a more appropriate model than Weibull, gamma, GHN and TN for this dataset because the sample quantiles are closer to the respective theoretical quantiles. Excepting the TPN distribution, all the other models present serious difficulties in accommodating the right tail of the data. Finally, the estimated skewness and kurtosis coefficients for the TPN model consider that the MLEs are 0.694 and 3.731. The 95% confidence intervals (CI) for those coefficients estimated via bootstrap (based on 10,000 bootstrap samples) are given by (0.167; 1.420) and (2.411; 6.841), respectively. Note that the sample versions of both coefficients are contained in the estimated CI.

### 5.2. Breaking Stress of Carbon Fibers

This dataset is considered in [31] and corresponds to breaking stress of carbon fiber (BSFC) measures in Gba. Cordeiro and Lemonte [26] already analyzed these data comparing the BS and β-BS models. Additionally, we also compared those models with the EHN and PHN distributions. Table 5 presents basic descriptive statistics for the dataset. Note that for this dataset, the sample minimum is close to zero. Therefore, in this case, it seems reasonable to consider c=0.

We also computed the moment estimators, resulting in ${\hat{\sigma }}_{M}=1.604$ and ${\hat{\alpha }}_{M}=14.505$, which were used as initial estimates for the maximum likelihood approach.

**Figure 8:**
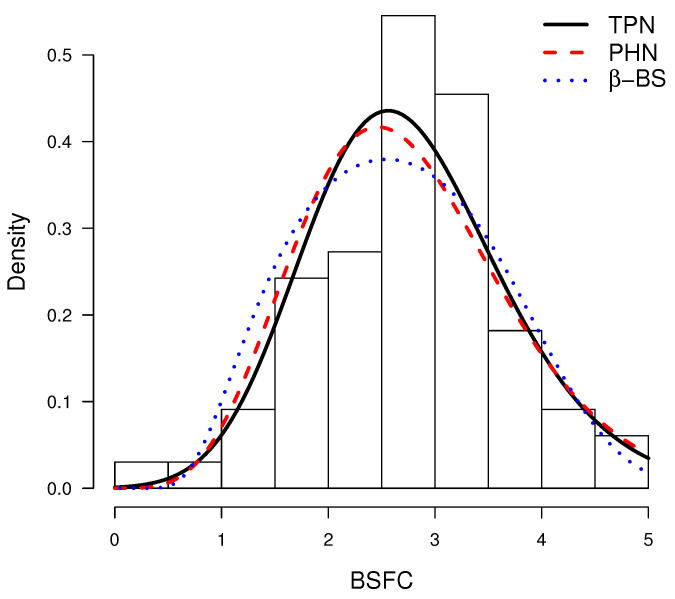
Histogram for the BSFC dataset, with lines representing adjusted distributions using MLE using different models.

**Figure 9:**
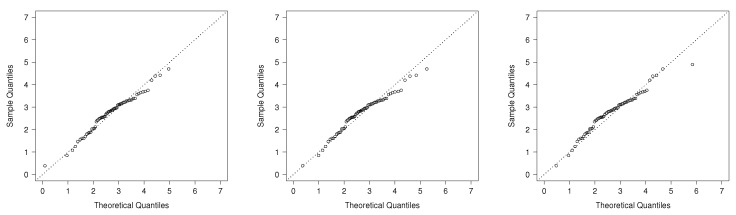
q-q plots: TPN model (**left**), PHN model (**center**) and β-BS model (**right**).

Table 6 shows the MLEs. It can be noted that AIC shows a better fit of the TPN model. In Figure 8, the ML setting of models is shown with the probability histogram. Finally, from the q-q plots in Figure 9, we have that the TPN model fits the data better than the other models considered.

## 6. Discussion

The main focus of this paper is studying a truncated positive version of the PN model, obtaining a new extension of the HN model. This model involves two parameters and is an alternative to other positive models. Maximum likelihood estimation is conducted for parameter estimation, and results of a simulation study indicate that it has good properties for small and moderate sample sizes, as well as applications to real data, indicating that it can outperform competing distributions. A simulation study was implemented using the acceptance rejection method for some truncation values, and the results were satisfactory.

## Figures and Tables

**Table 1:** Mean of the estimated parameters (mean), mean of the estimated standard deviations (s.d.) and root of the mean squared error ($\sqrt{MSE}$) for MLEs of the $TPN_{c}\left(\sigma ,\alpha \right)$ model (cases n=30 and n=50).

True Value			n=30						n=50					
			$\hat{\alpha }$			$\hat{\sigma }$			$\hat{\alpha }$			$\hat{\sigma }$		
c	α	σ	mean	s.d.	$\sqrt{\mathrm{MSE}}$	mean	s.d.	$\sqrt{\mathrm{MSE}}$	mean	s.d.	$\sqrt{\mathrm{MSE}}$	mean	s.d.	$\sqrt{\mathrm{MSE}}$
0.0	0.8	1	1.235	1.099	1.221	0.959	0.164	0.166	1.304	1.050	0.987	0.953	0.136	0.133
		2	1.238	1.097	1.221	1.923	0.329	0.328	1.229	1.018	0.968	1.923	0.274	0.268
		3	1.244	1.095	1.234	2.876	0.491	0.499	1.137	0.935	0.964	2.922	0.406	0.41
	1.0	1	1.414	1.145	1.269	0.965	0.164	0.169	1.399	1.052	0.951	0.964	0.136	0.13
		2	1.420	1.159	1.262	1.932	0.329	0.330	1.352	1.023	0.971	1.943	0.273	0.262
		3	1.412	1.156	1.280	2.901	0.494	0.502	1.286	0.960	0.980	2.934	0.404	0.405
	1.5	1	1.857	1.265	1.353	0.975	0.163	0.162	1.770	1.056	0.996	0.980	0.132	0.126
		2	1.835	1.261	1.336	1.955	0.326	0.328	1.721	1.043	0.992	1.972	0.265	0.255
		3	1.838	1.259	1.348	2.936	0.490	0.495	1.695	1.016	1.030	2.966	0.396	0.39
0.5	0.8	1	2.256	2.383	3.004	0.945	0.149	0.157	2.423	2.421	2.441	0.932	0.129	0.131
		2	1.595	1.568	1.866	1.907	0.314	0.324	1.687	1.538	1.497	1.891	0.265	0.264
		3	1.468	1.394	1.605	2.863	0.477	0.490	1.355	1.219	1.241	2.897	0.398	0.406
	1.0	1	2.329	2.415	2.996	0.953	0.151	0.155	2.537	2.418	2.451	0.936	0.128	0.131
		2	1.730	1.607	1.888	1.921	0.315	0.325	1.801	1.544	1.498	1.901	0.265	0.262
		3	1.615	1.454	1.634	2.884	0.482	0.497	1.507	1.249	1.261	2.911	0.398	0.402
	1.5	1	2.665	2.553	2.989	0.965	0.153	0.154	2.764	2.388	2.313	0.953	0.128	0.122
		2	2.163	1.770	1.965	1.939	0.318	0.320	2.088	1.542	1.468	1.937	0.262	0.251
		3	2.020	1.570	1.714	2.922	0.483	0.480	1.858	1.290	1.294	2.949	0.394	0.385
1.0	0.8	1	8.602	10.109	12.768	0.912	0.146	0.171	3.867	4.601	6.288	0.953	0.112	0.117
		2	2.287	2.389	3.112	1.891	0.298	0.317	2.378	2.381	2.455	1.868	0.257	0.263
		3	1.733	1.777	2.139	2.858	0.465	0.479	1.846	1.740	1.765	2.830	0.392	0.4
	1.0	1	8.637	10.050	12.698	0.914	0.146	0.169	4.061	4.658	6.454	0.956	0.112	0.118
		2	2.364	2.433	3.008	1.901	0.301	0.315	2.449	2.373	2.369	1.881	0.257	0.257
		3	1.963	1.866	2.227	2.866	0.466	0.481	1.942	1.743	1.734	2.855	0.393	0.393
	1.5	1	8.611	9.785	11.740	0.922	0.147	0.165	4.376	4.830	6.260	0.961	0.114	0.116
		2	2.664	2.565	2.986	1.932	0.307	0.313	2.754	2.374	2.354	1.907	0.256	0.247
		3	2.300	1.987	2.252	2.903	0.471	0.471	2.245	1.757	1.689	2.892	0.390	0.373

**Table 2:** Mean of the estimated parameters (mean), mean of the estimated standard deviations (s.d.) and root of the mean squared error ($\sqrt{MSE}$) for MLEs of the $TPN_{c}\left(\sigma ,\alpha \right)$ model (cases n=100 and n=200).

True Value			n=30						n=50					
			$\hat{\alpha }$			$\hat{\sigma }$			$\hat{\alpha }$			$\hat{\sigma }$		
c	α	σ	mean	s.d.	$\sqrt{\mathrm{MSE}}$	mean	s.d.	$\sqrt{\mathrm{MSE}}$	mean	s.d.	$\sqrt{\mathrm{MSE}}$	mean	s.d.	$\sqrt{\mathrm{MSE}}$
0.0	0.8	1	0.967	0.707	0.678	0.986	0.102	0.100	0.857	0.508	0.511	0.996	0.074	0.074
		2	0.923	0.675	0.690	1.979	0.202	0.199	0.856	0.508	0.511	1.99	0.149	0.147
		3	0.930	0.672	0.687	2.964	0.301	0.297	0.850	0.509	0.505	2.989	0.223	0.221
	1.0	1	1.129	0.719	0.701	0.991	0.101	0.100	1.046	0.519	0.524	0.998	0.073	0.073
		2	1.107	0.701	0.712	1.985	0.200	0.197	1.040	0.519	0.522	1.996	0.146	0.145
		3	1.104	0.705	0.706	2.976	0.301	0.297	1.042	0.518	0.521	2.994	0.22	0.218
	1.5	1	1.591	0.739	0.735	0.995	0.096	0.096	1.535	0.525	0.529	0.999	0.069	0.069
		2	1.584	0.735	0.746	1.990	0.192	0.191	1.545	0.525	0.525	1.994	0.137	0.138
		3	1.566	0.735	0.738	2.990	0.289	0.284	1.532	0.525	0.53	2.998	0.206	0.209
0.5	0.8	1	1.287	1.252	1.402	0.979	0.092	0.092	1.058	0.931	0.992	0.989	0.069	0.068
		2	1.057	0.907	0.961	1.968	0.193	0.192	0.915	0.681	0.705	1.988	0.144	0.143
		3	1.003	0.816	0.853	2.957	0.293	0.290	0.882	0.622	0.621	2.984	0.22	0.215
	1.0	1	1.435	1.304	1.424	0.983	0.093	0.091	1.202	0.967	1.020	0.993	0.069	0.069
		2	1.195	0.944	0.974	1.980	0.195	0.190	1.095	0.714	0.724	1.993	0.145	0.142
		3	1.178	0.860	0.883	2.969	0.295	0.293	1.068	0.644	0.641	2.992	0.219	0.216
	1.5	1	1.850	1.432	1.513	0.991	0.094	0.093	1.656	1.058	1.095	0.997	0.07	0.069
		2	1.639	1.022	1.041	1.991	0.194	0.192	1.560	0.748	0.755	1.998	0.141	0.141
		3	1.637	0.921	0.930	2.984	0.290	0.287	1.563	0.666	0.673	2.994	0.209	0.209
1.0	0.8	1	2.469	2.839	3.670	0.972	0.084	0.086	2.445	2.674	2.689	0.967	0.068	0.066
		2	1.287	1.265	1.403	1.960	0.185	0.183	1.044	0.938	0.981	1.980	0.138	0.134
		3	1.102	1.001	1.067	2.953	0.285	0.282	0.963	0.759	0.778	2.975	0.213	0.207
	1.0	1	2.586	2.954	3.590	0.974	0.085	0.086	2.542	2.683	2.666	0.971	0.068	0.066
		2	1.439	1.323	1.434	1.967	0.186	0.186	1.224	0.982	1.015	1.984	0.139	0.138
		3	1.290	1.064	1.121	2.959	0.288	0.283	1.145	0.796	0.824	2.979	0.214	0.213
	1.5	1	2.962	3.118	3.723	0.979	0.086	0.086	2.810	2.701	2.610	0.976	0.069	0.064
		2	1.844	1.429	1.509	1.982	0.188	0.186	1.658	1.063	1.069	1.993	0.14	0.136
		3	1.704	1.147	1.178	2.980	0.289	0.284	1.597	0.841	0.848	2.994	0.211	0.211

**Table 3:** Descriptive statistics.

Dataset	*n*	$\bar{X}$	*S*	$\sqrt{b_{1}}$	$b_{2}$	min(x)	max(x)
BMI	202	22.96	8.20	0.95	5.18	16.75	34.42

**Table 4:** Parameter estimates (with their respective standard deviations in parenthesis) and AIC values for Weibull, Gamma, generalized half-normal (GHN) and TPN models.

Estimates	Weibull	Gamma	GHN	TPN	TPN	TN
σ	24.259(0.249)	0.339(0.034)	24.954(0.283)	7.667(0.225)	7.676 (0.229)	1.050 (0.142)
α	7.281(0.340)	67.804(6.730)	4.949(0.070)	439.234(109.204)	433.809 (109.878)	-
λ	-	-	-	-	-	8.035 (0.406)
*c*	-	-	-	0 (-)	16 (-)	-
log-likelihood	−524.52	−492.73	−545.91	−488.98	−488.93	−498.67
AIC	1053.04	989.45	1095.81	981.97	981.85	1001.33

**Table 5:** Descriptive statistics. BSFC, breaking stress of carbon fiber.

Data Set	*n*	$\bar{X}$	*S*	$\sqrt{b_{1}}$	$b_{2}$	min(x)	max(x)
BSFC	66	2.760	0.891	−0.13	3.22	0.39	4.90

**Table 6:** Parameter estimates (with their respective standard deviations in parenthesis) and AIC values for the Birnbaum–Saunders (BS), β-BS, epsilon half-normal (EHN) and TPN models.

Estimates	BS	β-BS	EHN	TPN	PHN
σ	-	-	2.898(0.252)	1.679(0.101)	0.570 (0.118)
α	0.437(0.038)	1.045(0.004)	0.003(0.068)	12.470(2.252)	1.581 (0.913)
β	2.515(0.132)	57.600(0.331)	-	-	-
*a*	-	0.193(0.026)	-	-	-
*b*	-	1876.732(605.050)	-	-	-
log-likelihood	−100.19	−91.35	−118.13	−87.21	−89.18
AIC	204.38	190.71	240.25	178.42	182.37
